# Combination therapy with ropivacaine-loaded liposomes and nutrient deprivation for simultaneous cancer therapy and cancer pain relief

**DOI:** 10.7150/thno.43932

**Published:** 2020-03-26

**Authors:** Jiqian Zhang, Shasha Zhu, Qilian Tan, Dan Cheng, Qingqing Dai, Zhilai Yang, Lei Zhang, Fenfen Li, Youmei Zuo, Wei Dai, Lihai Chen, Erwei Gu, Guanghong Xu, Zhaolian Wei, Yunxia Cao, Xuesheng Liu

**Affiliations:** 1 Department of Anesthesiology, the First Affiliated Hospital of Anhui Medical University, Key Laboratory of Anesthesia and Perioperative Medicine of Anhui Higher Education Institutes; 2 Reproductive Medicine Center, Department of Obstetrics and Gynecology, the First Affiliated Hospital of Anhui Medical University, Anhui Province Key Laboratory of Reproductive Health and Genetics, Biopreservation and Artificial Organs, Anhui Provincial Engineering Research Center, Anhui Medical University; 3 Department of Anesthesiology, Nanjing First Hospital, Nanjing Medical University; 4 Centre for Biomedical Engineering, Department of Electronic Science and Technology, University of Science and Technology of China

**Keywords:** autophagy, cancer, cancer pain, VEGF-A, STAT3, ropivacaine, liposome

## Abstract

Autophagy allows cancer cells to respond changes in nutrient status by degrading and recycling non-essential intracellular contents. Inhibition of autophagy combined with nutrient deprivation is an effective strategy to treat cancer. Pain is a primary determinant of poor quality of life in advanced cancer patients, but there is currently no satisfactory treatment. In addition, effective treatment of cancer does not efficiently relieve cancer pain, but may increase pain in many cases. Hence, few studies focus on simultaneous cancer therapy and pain relief, and made this situation even worse.

**Method**: Ropivacaine was loaded into tumor-active targeted liposomes. The cytotoxicity of ropivacaine-based combination therapy in B16 and HeLa cells were tested. Moreover, a mice model of cancer pain which was induced by inoculation of melanoma near the sciatic nerve was constructed to assess the cancer suppression and pain relief effects of ropivacaine-based combination therapy.

**Results**: Ropivacaine and ropivacaine-loaded liposomes (Rop-DPRL) were novelly found to damage autophagic degradation. Replicated administration of Rop-DPRL and calorie restriction (CR) could efficiently repress the development of tumor. In addition, administration of Rop-DPRL could relieve cancer pain with its own analgestic ability in a short duration, while repeated administration of Rop-DPRL and CR resulted in continuous alleviation of cancer pain through reduction of VEGF-A levels in advanced cancer mice. Further, dual inhibition of phosphorylation of STAT3 at Tyr705 and Ser727 by Rop-DPRL and CR contribute to the reduction of VEGF-A.

**Conclusion**: Combination therapy with Rop-DPRL and nutrient deprivation simultaneously suppresses cancer growth and relieves cancer pain.

## Introduction

Autophagy is a conserved lysosome-based dynamic intracellular degradative process that plays an important role in regulating cellular homeostasis and responding to changes in nutrient status 1, 2. During starvation, autophagosomes engulf cytoplasmic constituents and fuse with lysosomes to form autolysosomes, which subsequently degrade the engulfed contents to provide nutrients to starved cells and promote cell survival 3-5. Compared with normal cells, cancer cells undergo uncontrolled and rapid proliferation, resulting in extreme sensitivity to nutrient deprivation 6. As such, disruption of autophagy combined with nutrient deprivation is considered as a potentially effective approach to treat cancer 7.

Pain is a common symptom associated with many cancers. The frequency and intensity of pain increases with cancer progression. Approximately 75-95% of patients with advanced cancer experience cancer-related pain, including local hypersensitivity to mechanical and/or thermal stimuli, which severely affects their life quality 8. Cancer pain is a complex pain state involving inflammatory, neuropathic, compressive, and ischemic mechanisms 9. In the cancer microenvironment, algogenics mediators secreted by cancers cells can sensitize and activate nociceptors 10. Nerve growth factor (NGF) and vascular endothelial growth factor (VEGF) are two representative mediators secreted by cancer cells that contribute to hyperinnervation of pain-mediating nerve fibers in cancer tissues 10, 11. The VEGF protein family selectively activate VEGFR1, which is expressed in nociceptors, leading to increased nociceptor excitability and pain hypersensitivity 11. VEGF-A, as a specific ligand for VEGFR1, was reported to be critical in tumor angiogenesis 12. Constitutive signal transducer and activator of transcription 3 (STAT3) activity can induce VEGF-A transcription, and resulting in angiogenesis 13. The mammalian target of rapamycin (mTOR) kinase acts in two functionally distinct complexes, mTOR complex 1 (mTORC1) and mTOR complex 2 (mTORC2)^14^. mTORC1 promotes STAT3 activation through direct phosphorylation of Ser727. STAT3 is also regulated by phosphorylation of Tyr705 through a number of different signaling pathways activated by cytokines and growth factors, such as IL-6, which signal through the receptor tyrosine kinase JAK2 15.

Effective treatment of cancer does not efficiently relieve cancer pain, but may increase pain in many cases. Then, opioid analgesics gradually evolve into the gold standard treatment for cancer-related pain. However, the opioid use is associated with some side effects, such as nausea, vomiting, and habituation 16. Thus, new strategies for treating cancer pain are urgently needed. Local anesthetics are a class of drugs, which have wide applications in the treatment of acute and chronic pain. But their analgesic effects only last for a few hours because of short half-life, which limits their applications in treating cancer pain 17. However, encapsulation of local anesthetics into lipid-based nanocarriers presents advantages such as slow release, prolonged duration of action and low toxicity to the central nervous and cardiovascular systems 18. These may provide the possibility of treating cancer pain using local anesthetics.

Lipid-based nanodrugs are broadly reported in relieving inflammatory pain 19, neuropathic pain 20, 21, rheumatoid arthritis 22 and prolonging synergistic analgesia 23. In addition, taking the advantages of nanotechnology, analgesic microneedle patch 24 and near-infrared light-sensitive nano neuro-immune blocker capsule 25 were suggested to relief neuropathic pain. Gold half-shell multifunctional nanoparticles were also reported to treat rheumatoid arthritis 26. Nonetheless, few nanodrugs are reported to treat cancer pain. Additionally, nanosized drug-delivery platforms can improve the bioavailability of drugs by sustained drug release and avoid removal by the reticuloendothelial system (RES) 27. Furthermore, drugs can be enhanced by passively targeting tumors based on the enhanced permeability and retention (EPR) effect or active targeting, simultaneously attenuating systemic toxicity 28, 29. As such, nano drugs are excellent candidates for the simultaneous treatment of cancer and its related pain.

**Figure 1:**
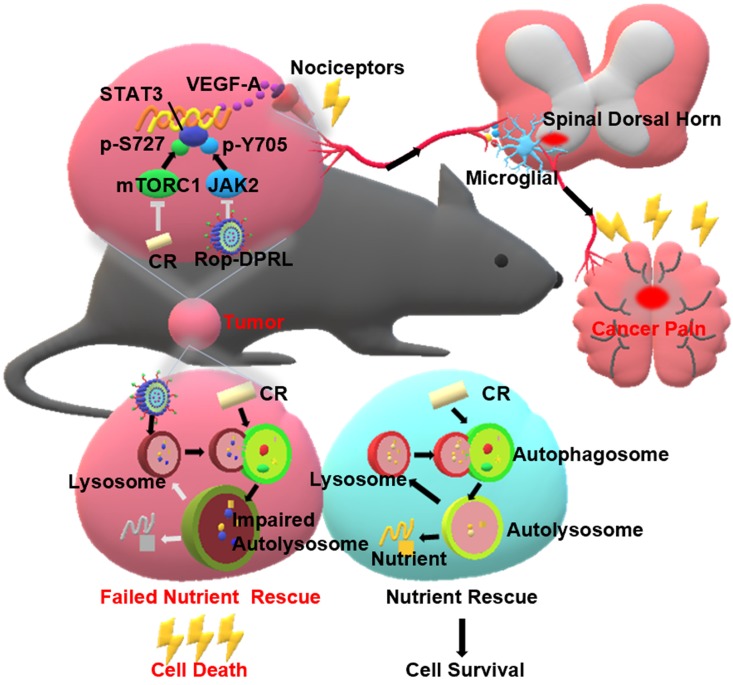
** A schematic illustration to show Rop-DPRL combined with CR simultaneously repress cancer growth and relieve cancer pain.** Rop-DPRL impairs autophagic degradation, thereby promoting cancer cell death and repressing development of cancer under CR condition. Rop-DPRL and CR inhibit phosphorylation of STAT3 at Tyr705 and Ser727 through the JAK2 and mTORC1 pathways, respectively. Inhibition of STAT3 activity at multiple sites reduces the secretion of algogenic mediator VEGF-A in tumor, leading to decreased number of activated microglia in the spinal dorsal horn and relief of cancer pain.

Ropivacaine is used clinically as a regional anesthetic, but its role in repressing cancer and alleviating cancer-related pain is rarely tested 30, 31. Malignant melanoma is the deadliest form of skin cancer with high recurrence and metastasis potential 32. Cervical cancer is one of the most common female malignancies around the world 33. Here, we tested the cytotoxicity of ropivacaine-based combination therapy in B16 and HeLa cells, which are representative cell lines of melanoma and cervical cancer, respectively^32^, ^34^. We novelly discovered that ropivacaine and ropivacaine-loaded liposome (Rop-DPRL) could damage autophagic degradation and promote the cytotoxicity of nutrient deprivation to cancer cells. Calorie restriction is a commonly used mean for nutrient deprivation in animal experiments. Replicated administration of Rop-DPRL and CR could efficiently repress the development of melanoma *in vivo*. As melanoma is often used in the study of different types of cancer pain 35-37, we constructed a mice model of cancer pain by inoculation of melanoma near the sciatic nerve 38 to assess the pain relief effect of ropivacaine-based combination therapy. The results indicated that administration of Rop-DPRL could relieve cancer pain with its own analgestic ability in a short duration, while repeated administration of Rop-DPRL and CR resulted in continuous alleviation of cancer pain through reduction of VEGF-A levels in advanced cancer mice. However, dox and cisplatin treatment only have anticancer effect but no analgesic effect. Furthermore, Rop-DPRL and CR inhibited phosphorylation of STAT3 at Tyr705 and Ser727 respectively, and this synergetic inhibiting effect account for VEGF-A reduction. These results suggest a new approach for simultaneously treating cancer and relieving cancer pain (Figure 1).

## Materials and Methods

### Antibodies and agents

Anti-phospho-STAT3 (S727) (1:100, ab32143), anti-phospho-STAT3 (Y705) (1:1000, ab76315), anti-VEGF-A (1:1000, ab1316), and anti-SQSTM1/P62 (1:2000, ab56416) were purchased from Abcam. Anti-phospho-p70S6 kinase (1:1000, 9234) and anti-phospho-JAK2 (1:1000, 4406) were purchased from Cell Signaling Technology. Anti-LC3 (1:2000, NB100-2220) was purchased from Novus Biologicals. Anti-β-actin (1:1000, 60008-1-Ig), anti-JAK2 (1:1000, 17670-1-AP), anti-STAT3 (1:1000, 60199-1-Ig), anti-p70s6k (1:1000, 14485-1-AP), and anti-TSC1 (1:1000, 20988-1-AP) were purchased from Proteintech. Anti-cathepsin D (1:1000, sc-6486) and anti-LAMP1 (1:1000, sc-71489) were purchased from Santa Cruz Biotechnology. HRP-conjugated anti-rabbit antibody (W4011), HRP-conjugated anti-goat antibody (V805A), and HRP-conjugated anti-mouse antibody (W4021) were purchased from Promega. Recombinant VEGF-A was purchased from Sino Biological. Apoptosis and Necrosis Assay Kit was purchased from Beyotime Biotechnology, enhanced chemiluminescence (ECL) kits were purchased from Biological Industries, TUNEL Bright Red Apoptosis Detection Kit (A113-01) was purchased from Vazyme, and Lipofectamine™ 2000 Transfection Reagent was purchased from Thermo Fisher Scientific. Lecithin was purchased from Aladdin (L105732) and DSPE-PEG2000-RGD was purchased from Ruixi biological technology (R-9958). Chloroquine (CQ, PHR1258) and cholesterol (C8667) was purchased from Sigma-Aldrich, ropivacaine was purchased from MACKLIN (R832515).

### Preparation and Characterization of Rop-DPRL

Liposomes were obtained using the thin-film hydration method. Briefly, Phospholipids (Lecithin, cholesterol and DSPE-PEG2000-RGD at a molar ratio 66:30:4) were dissolved and mixed in chloroform/ methanol (3:1 v/v). Then, the phospholipids mixture was thoroughly dried with a rotary evaporator to form a lipid film. Lipid film were dissolved in PBS or 30 mg/mL ropivacaine solution to obtain empty liposomes or Rop-DPRL, respectively. The liposome dispersion was then extruded through a 0.1 µm pore-size polycarbonate filter (Whatman, 131125012E) to obtain uniform particles. The morphology of Rop-DPRL was characterized by transmission electron microscope (JEOL- 2010). The particle size distribution and zeta potential of Rop-DPRL were determined using dynamic light scattering (DLS) with particle size analyzer (90 Plus, Brookhaven Instruments Co.).

### *In Vitro* Release Study

Using the dialysis method with a membrane of a molecular weight cutoff of 3500 kDa, the *in vitro* drug release test was conducted. 1 mL of Rop-DPRL solution was placed into a dialysis bag and then was placed in the PBS at 37 °C, followed by shaking at 100 rpm. At specified time points, 1 mL of the dissolution media was collected and supplemented with the same amount of fresh dialysis fluid. The amount of ropivacaine released was measured using HPLC (Agilent 6890).

### Cell culture

Cells were cultured at 37 °C with 5% CO_2_ in Dulbecco's Modified Eagle's Medium supplemented with 10% fetal bovine serum (FBS). Cells were starved by incubation in serum-free DMEM without glutamine 3. GFP-LC3-HeLa and B16 were provided by Prof. Longping Wen from the South China University of Technology.

### Lysosome marker dye staining

Cells were treated with 75 nM LysoTracker Red for 15 min, followed by two washes with PBS. The cells were visualized using a fluorescence microscope (Olympus IX71, Olympus, Japan).

### Statistical analysis of subcellular structures

Lysosomes were counted using Image J software with the “analyze particles-count/size” tool with default settings 39.

### Immunofluorescence

Cells were fixed using 4% paraformaldehyde for 10 min, permeabilized with 0.1% Triton X-100 for 10 min, and blocked with 1% FBS for 1 h. Cells were incubated with primary antibodies overnight at 4 °C and labeled with secondary antibodies at 37 °C for 1 h. Images were acquired using fluorescence microscopy (Olympus IX71, Olympus, Japan). Mice were anesthetized with sodium pentobarbital and perfused with PBS, followed by 4% paraformaldehyde. Following perfusion, the L4-L6 spinal cord segments were removed and post-fixed overnight in fixative solution, then cryoprotected overnight in 30% sucrose in PBS. Frozen spinal cord tissues were embedded in TissueTek OCT compound, then cut into 10 μm sections. The sections were evaluated for immunofluorescence.

### Western blotting

Cells were lysed with sample buffer and boiled for 10 min. Proteins were separated by sodium dodecylsulfate polyacrylamide gel electrophoresis and were transferred to nitrocellulose membranes. The membranes were incubated with primary antibodies at 4 °C overnight, then with secondary antibodies for 1 h at 37 °C. Membranes were incubated with ECL kit reagents and visualized using a chemiluminescence instrument (ImageQuant LAS 4000, GE Healthcare, Little Chalfont, UK).

### Cell viability assay

Cells were seeded in 96-well plates (10^4^ cells/well) and cultured at 37 °C with 5% CO_2_. 10 μL of 5 mg/mL MTT was added to each well and incubated at 37 °C for 4 h. After removing the medium, formazan crystals were dissolved in 100 μL of DMSO, and absorbance was measured at 570 nm using a microplate reader (Nano Quant, Tecan, Männedorf, Switzerland).

### Cell death assay

Cells were stained with Hoechst 33342 (5 μL) and propidium iodide (5 μl) (Apoptosis and Necrosis Assay Kit purchased from Beyotime Biotechnology) for 15 min and examined using a fluorescence microscope. Cell death was quantified by counting more than 600 cells in each group, and the results were expressed as the ratio of PI-positive cells to Hoechst-positive cells.

### Lentiviral transduction

Non-targeting shRNA, TSC1-targeting shRNA, JAK2 V617F, and empty vector with pMD2.G and pPsAX2.0 were transfected into HEK293T cells with Lipofectamine 2000. After 2 d, HeLa cells were infected with the filtered lentiviruses in medium containing 5 μg/mL polybrene. The transduced cells were selected with 1 μg/ml puromycin for 4 d. A pLKO1 shRNA construct against TSC1 was purchased from Public Protein/Plasmid Library with the following sequence: 5'-GATCCGCAGCCATCTTGGAAGCATAATTTCAAGAGAATTATGCTTCCAAGATGGCTGCTTTTTTG-3'.

### Animals

Six to eight weeks old male C57BL/ 6 mice, weighing 18-25 g, were purchased from the Model Animal Research Center of Nanjing University. All animals were housed in temperature, humidity, and light controlled rooms, with water provided ad libitum. Animal welfare and experimental procedures were carried out in accordance with the Ethical Regulations on the Care and Use of Laboratory Animals of Anhui Medical University and were approved by the school committee for animal experiments.

Mice were anesthetized with sodium pentobarbital. One million B16 cells in 100 mL of sterile PBS were injected into the muscular tissue in the immediate vicinity of the nerve near the trochanter 38. Seven days after inoculation, tumor diameters reached 4-6 mm 40, indicating establishment of the melanoma mouse model. The mice were randomly divided into 4 groups: 1) control (normally fed, i.p. injection of PBS every other day), 2) Rop-DPRL (i.p. injection of 64.4 mg/kg Rop-DPRL every other day), 3) CR (fed 70% of normal food intake, i.p. injection of PBS every other day), and 4) Rop-DPRL + CR (fed 70% of normal food intake, i.p. injection of 64.4 mg/kg Rop-DPRL every other day). Tumor sizes were measured daily. Tumor volume was calculated using the following formula: length × width^2^ / 2 = tumor volume (mm3) 41. When the tumor size neared 2000 mm^3^ 42, all mice were sacrificed and the excised tumors were weighed to evaluate antitumor effect. For dox or cisplatin treatment, mice received i.p. injections of dox (1.5 mg/kg, 5 d/wk) or cisplatin (4 mg/kg, 3 d/wk) once per day. For VEGF-A administration, 500 ng/kg VEGF-A was injected into the tumor tissue 3 h before measurement of mechanical hyperalgesia.

### Biodistribution assessment

Tumor-bearing BALB/c mice were intraperitoneal injected with Cy5 labelled Rop-DPRL (0.4 μmol/kg Cy5). Mice were sacrificed at 12 h or 24 h and major organs were excised for ex vivo imaging by SPECTRAL ami HT. Tumor tissue were made into frozen sections and were observed under confocal microscope (Zeiss LSM710).

### Behavioral assessment

Hind paw lifting was evaluated to assess mechanical withdrawal threshold. Mice were placed in plastic cages with wire-net floors. The rigid tip of a 2450 series electronic Von Frey aesthesiometer (IITC 2091) was placed onto the plantar surface of the hind paw and pressed upward slowly until a withdrawal reflex was observed, and the force that elicited the withdrawal reflex was recorded 38. For the thermal pain threshold determination on mice hind paw, the Hargreaves test was used with a paw thermal stimulator system (IITC 390), which applies a high-intensity beam of light directed to the hind paw to induce pain. The time it takes for the animal to withdraw its hind paw (withdrawal latency) was measured. A cutoff of 25 s is employed to avoid excessive tissue injury. Mechanical and thermal pain was tested at the 7th and 13th d after inoculation. For each test, baseline mechanical withdrawal threshold and thermal withdrawal latency levels, and their levels at 12 h and 24 h after Rop-DPRL injection were recorded.

### Statistical analysis

Distribution normality was assessed using the Kolmogorov-Smirnov test. Pain behavior and tumor volume were analyzed by one-way or two-way repeated measures analysis of variance with Turkey's post hoc test. Western blots, immunofluorescence assays and tumor weight were analyzed by one-way analysis of variance with Turkey's post hoc test or Kruskal-Wallis test. Statistical analysis was performed using GraphPad Prism 7.0 software.

## Results and Discussion

### Preparation and characterization of Rop-DPRL

**Figure 2:**
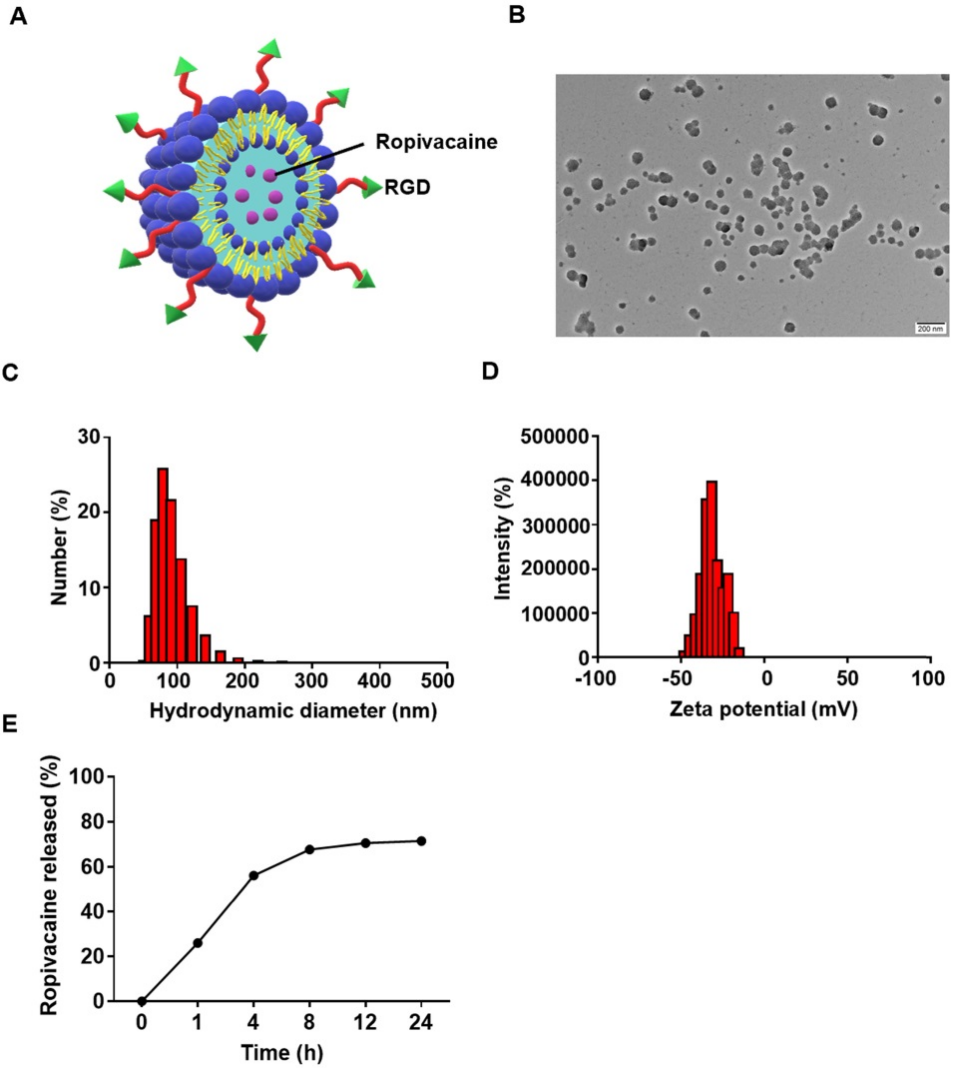
** Characterization of Rop-DPRL. (A)** Pattern diagram of Rop-DPRL. **(B)** TEM image of Rop-DPRL. **(C)** The average hydrodynamic size of Rop-DPRL suspended in PBS. **(D)** The zeta potential of Rop-DPRL suspended in PBS. **(E)** Cumulative release profile of Rop-DPRL.

Ropivacaine was loaded into liposomes which constituted by lecithins, cholesterol and DSPE-PEG2000-RGD (Figure 2A). RGD is a short peptide that is commonly used to actively target tumor cells to enhance drug delivery 43. The morphology was determined by TEM, which showed the particles have a uniform size (Figure 2B). The dynamic light scattering (DLS) analysis showed the hydrodynamic diameter of Rop-DPRL was 127.3 nm (Figure 2C) (polydispersity index, PDI, 0.071), and the ξ potential values was -27.8 mv (Figure 2D). It was reported that a liposome diameter of about 100 nm is likely to be an optimal size, not only for the more effective extravasation of liposomes, but also for their longer retention in tumor tissue 44. In addition, as shown in (Figure 2E), the accumulative release rate of Rop-DPRL was 71.4% after 24 h.

### Rop-DPRL impairs autophagy

**Figure 3:**
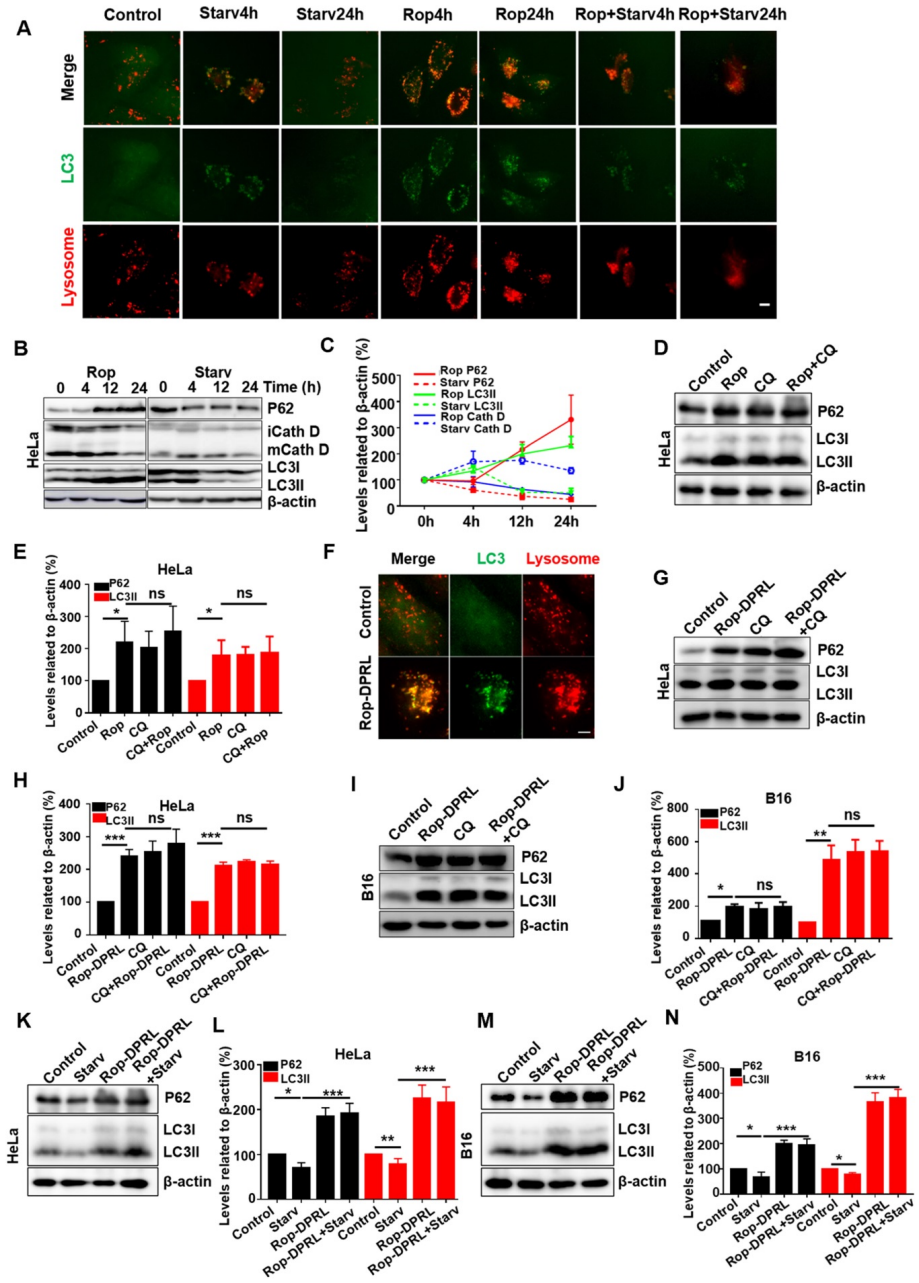
** Rop-DPRL impairs autophagy. (A)** Fluorescent images of GFP-LC3-HeLa cells treated with 1.5 mM ropivacaine for 0-24 h, or starvation for 0-24 h, and stained with 75 nM LysoTracker Red. Scale bar = 5 µm. **(B)** Western blot and **(C)** statistical results of P62, immature and mature cathepsin D (CathD), LC3I, LC3II, and β-actin. HeLa cells were treated with ropivacaine or starvation for 0-24 h. **(D)** Western blot and **(E)** statistical results of P62, LC3I, LC3II, and β-actin. HeLa cells were treated with PBS, CQ (50 µM), ropivacaine, or CQ+Rop for 24 h. **(F)** Fluorescent images of GFP-LC3-HeLa cells treated with empty liposome or Rop-DPRL for 24 h and stained with 75 nM LysoTracker Red. Scale bar = 5 μm. **(G,I, K and M)** Western blot and **(H,J,L and N)** statistical results for P62, LC3I, LC3II and β-actin. Cells were treated with empty liposome, CQ, Rop-DPRL, starvation or indicated combination for 24 h. Data are presented as the mean ± SEM, *p < 0.05, ** p < 0.01, *** p < 0.001, ns = no significant difference, Rop: ropivacaine, starv: starvation.

During nutrient deprivation, autophagosomes engulf cytoplasmic constituents and fuse with lysosomes to form autolysosomes, which subsequently degrade the engulfed contents to provide nutrients to starved cells and promote cell survival 3-5. Formation of autolysosomes results in consumption of lysosomes 5, 45. Consumption of lysosomes can be rescued by autophagic lysosome reformation (ALR) 45, 46, which is characterized by “budding” of tubular pro-lysosomes from autolysosomes, and subsequent maturation of these structures into lysosomes. LC3 is a marker protein of autophagosome and lysosome can be stained by LysoTracker Red. Structures with overlapping signal for lysosomes and LC3 were considered autolysosomes. In this study, nutrient deprivation was simulated by starvation treatment, in which serum and glutamine were extracted from the medium. Consistent with previous study 45, autolysosomes were formed at 4 h, and most had converted to lysosomes at 24 h in starved cells. In contrast, enlarged autolysosomes persisted in ropivacaine-treated cells (Figure 3A). Statistical analysis showed that the number of lysosomes was reduced at 4 h, and partially recovered at 24 h in starved cells; however, the number of lysosomes continued to decrease in ropivacaine-treated cells (Figure S1). These results indicated that ALR was disrupted by ropivacaine.

Autophagic degradation deficit is the most common cause of ALR disruption 47. Therefore, we examined autophagic degradation in ropivacaine-treated cells. Sequestosome 1 (SQSTM1/P62), a protein substrate that is selectively incorporated into autophagosomes and degraded by autophagy 48, was evaluated, with starvation as a positive control. As shown in Figure 3B-C, P62 levels were decreased in starved cells, but increased in ropivacaine-treated cells, which suggested impaired degradation of P62. LC3 is cleaved from LC3I into a lower molecular weight LC3II and aggregates on to autophagosome membranes during the autophagy process. LC3 II has been shown to be degraded for recycling during the late stages of autophagy, resulting in decreased levels 49, 50. However, ropivacaine treatment resulted in continuous increased LC3 II levels (Figure 3B-C). Moreover, mature forms of the lysosomal protease cathepsin D (Cath D) 51 were decreased in ropivacaine-treated cells, but not in starved cells (Figure 3B-C), further indicating disruption of autolysosome function. To further characterize this effect, the autophagy inhibitor chloroquine (CQ), which acts by alkalizing lysosomes 52 was used. As shown in Figure 3D-E, additional treatment with CQ did not further increase the levels of P62, LC3 in ropivacaine-treated cells, revealing that autophagic degradation was impaired by ropivacaine.

To avoid the shortcomings of ropivacaine, we loaded it into tumor active-targeted liposomes (Rop-DPRL). Next, part of previous experiments was replicated in Rop-DPRL treated HeLa and B16 cells. As shown in Figure 3F, Rop-DPRL induced enlarged autolysosomes in HeLa cells. In addition, Rop-DPRL increased the levels of P62 and LC3II in HeLa and B16 cells, additional treatment with CQ did not further increase the levels of P62 and LC3II (Figure 3G-J). These results well confirmed the disruption of autophagic degradation by Rop-DPRL.

To achieve uncontrolled and rapid proliferation, cancer cells need masses of nutrients and energy. mTOR is a crucial signaling node that integrates environmental cues to regulate cell nutrients metabolism and protein synthesis, therefore mTOR is frequently hyper-activated in many types of cancers 14, 53. Nutrients deprivation inhibits mTOR activity and benefits cancer suppression 6. However, to resist nutrients deprivation, cancer cells may upregulate autophagy to promote nutrients supply and survival when mTOR signaling was inhibited. Hence, disruption of autophagy may stimulate the anticancer effect of nutrient deprivation 7. Thus, we detected whether autophagy is impaired by Rop- DPRL under starvation. As shown in Figure 3K-N, starvation decreased the levels of P62 and LC3II at 24h, however, additional Rop-DPRL treatment not only rescued but also further increased levels of P62 and LC3II, demonstrating the blockage of autophagy by Rop-DPRL under starvation.

### Rop-DPRL combined with starvation promotes cancer cells death

**Figure 4:**
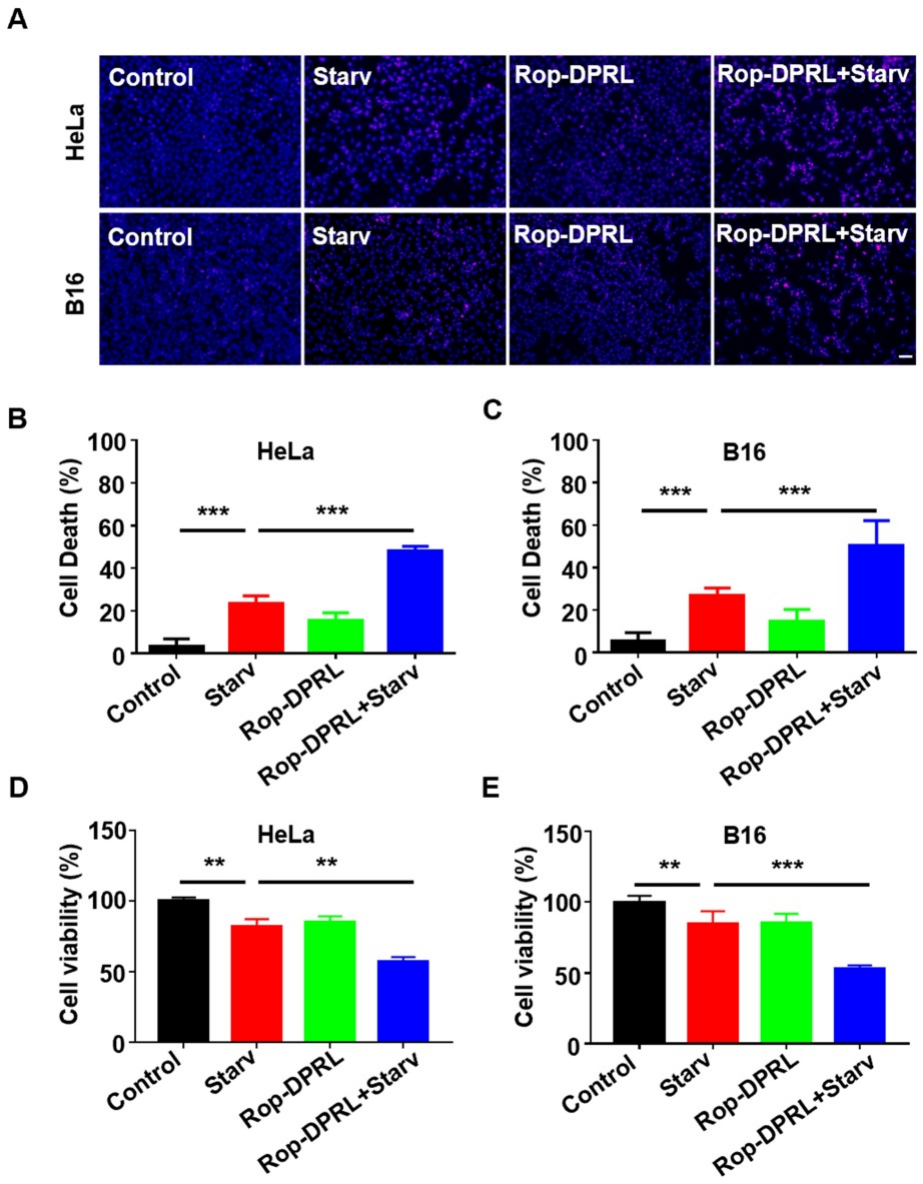
** Rop-DPRL promotes death of starved cancer cells. (A)** PI staining of HeLa or B16 cells. Nuclei were stained with Hoechst. Scale bar = 50µm. **(B, C)** Cells from (A) were analyzed for cell death percentage. **(D, E)** Cell viability was determined by MTT assay. HeLa or B16 cells were treated with empty liposome, starvation, Rop-DPRL or indicated combination for 24 h. Starv: starvation. Data are presented as the mean ± SEM, *p < 0.05, ** p < 0.01.

As nutrient deprivation is well known stimulus for induction of protective autophagy (Figure S2), while Rop-DPRL is found to inhibit autophagy, we hypothesize that Rop-DPRL will stimulate anticancer effect of nutrient deprivation. Indeed, starvation treatment resulted in death of 23.2% and 26.8% of HeLa and B16 cells, respectively. However, co-treatment with Rop-DPRL increased cell death of HeLa and B16 cells to 48.1% and 50.2%, respectively (Figure 4A-C). Trypan blue staining assessment showed a similar result (Figure S3). Furthermore, MTT results showed that combination treatment with Rop-DPRL and starvation significantly reduced cell viability compared to either treatment alone (Figure 4D-E). These results demonstrated that Rop-DPRL combined with starvation could significantly promote death of cancer cells.

### Rop-DPRL combined with CR inhibits tumor growth

**Figure 5:**
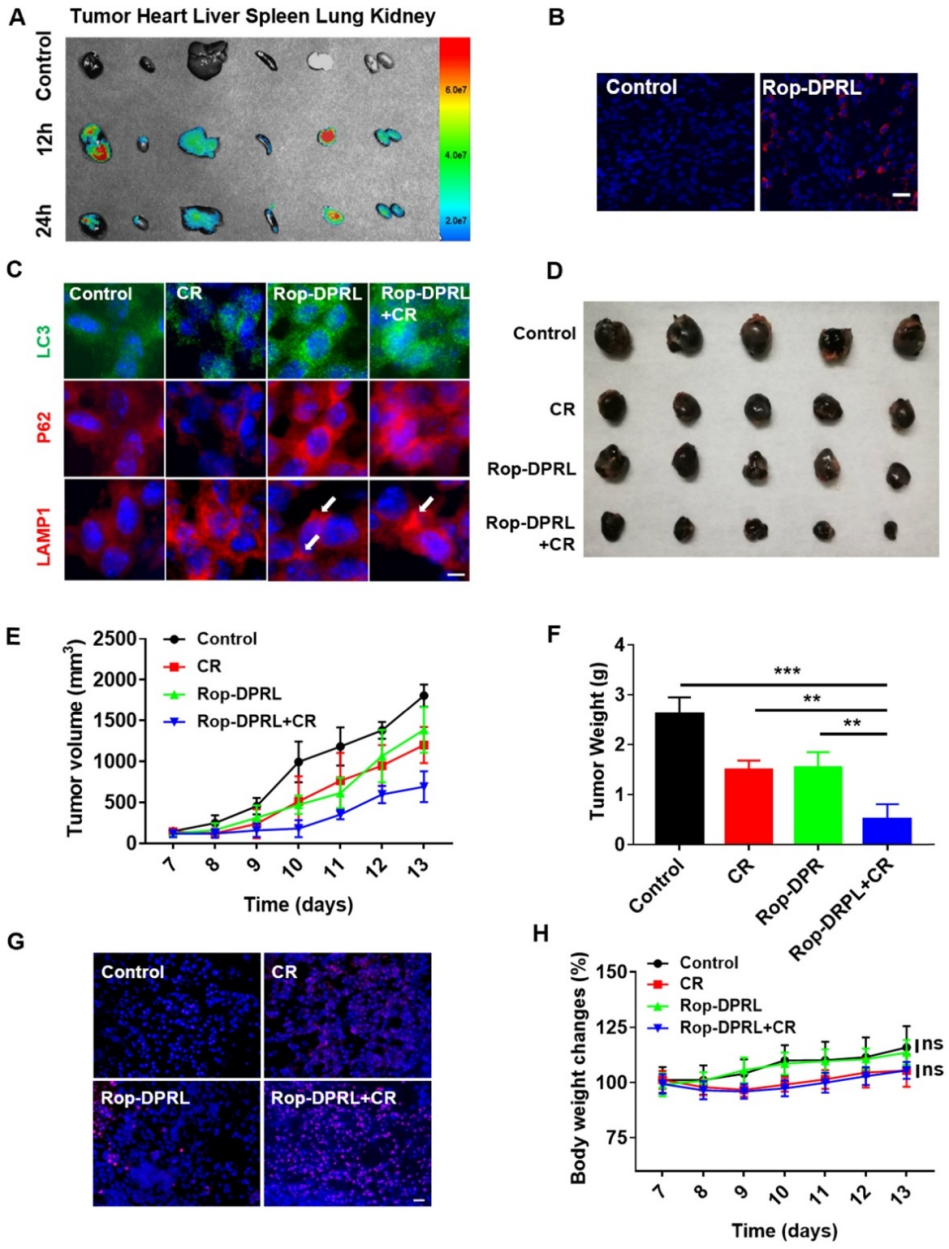
** Rop-DPRL combined with CR inhibits tumor growth. (A)** Fluorescent imaging of major organs and tumor. Tumor-bearing mice were injected with cy5-labeled liposome or unlabelled liposome. The tumor and major organs from control or drug-treated mice were excised at 12 h or 24 h. **(B)** Fluorescent image of tumor sections. Tumor from control or Rop-DPRL treated mice were excised at 12 h. Tumor frozen sections were observed under confocal microscope. Scale bar = 20 µm. **(C)** Immunofluorescence staining of tumor sections for the autophagosome marker LC3, P62 and LAMP1. **(D)** Image of representative tumors. **(E)** Change in tumor volume. **(F)** Tumor weight. **(G)** TUNEL staining of tumor sections. Nuclei were stained with Hoechst. Scale bar = 20µm. **(H)** Body weight changes of mice under different treatments. CR: Calorie restriction. Arrows: Enlarged lysosomes. Data are presented as the mean ± SEM, ** p < 0.01.

To further investigate the anticancer and analgesic effects of Rop-DPRL *in vivo*, a mouse model inoculated with B16 cells near the sciatic nerve was used 38, 54 in this study. Rop-DPRL obviously prolonged the residence time of ropivacaine in the blood and tissues (Figure S4). After 12 h of administration, many Rop-DPRL accumulated in tumor tissues (Figure 5A-B), and lots of Rop-DPRL were also detected in lung, liver and kidney (Figure 5A). Furthermore, the *in vivo* anticancer effect of Rop-DPRL was tested. Calorie restriction is a commonly used mean for nutrient deprivation in animal experiments 7. Tumor-bearing mice were randomly grouped and received treatments with empty liposome, CR alone, Rop-DPRL alone, or Rop-DPRL combined with CR. Rop-DPRL treatment increased P62 and LC3 levels in tumor tissues (Figure 5C), demonstrating the disruption of autophagy by Rop-DPRL *in vivo*. Enlarged lysosomes were also observed under Rop-DPRL treatment (Figure 5C), indicating abnormal autophagic/lysosomal degradation^47^, ^55^, ^56^. Notably, the overall LC3 levels were decreased but LC3 puncta were increased in CR mice. Daily tumor measurement showed mild inhibition of tumor growth by CR alone. However, treatment with Rop-DPRL combined with CR resulted in strong antitumor effect (Figure 5D-E). In addition, tumor size and weight were significantly lower in response to combination treatment compared with those in response to any single treatment (Figure 5E-F). Furthermore, TUNEL assay showed obvious tumor cells apoptotic following Rop-DPRL combined with CR treatment (Figure 5G). These results indicated that the combination therapy with ropivacaine and CR had a strong antitumor effect.

Moreover, we assessed the biosafety of Rop-DPRL based therapy. CR slightly decreased the mice body weight, and Rop-DPRL has no effect on the body weight and blood glucose levels both in CR mice and in normal diet mice (Figure 5H, Figure S5A). Besides, the physiological structures of the main organs were not affected by CR, Rop-DPRL and their combination therapy (Figure S5B). Collectively, Rop-DPRL combined with CR is a relatively safe therapeutic strategy.

### Rop-DPRL combined with CR results in continuous and specific relief of cancer pain

**Figure 6:**
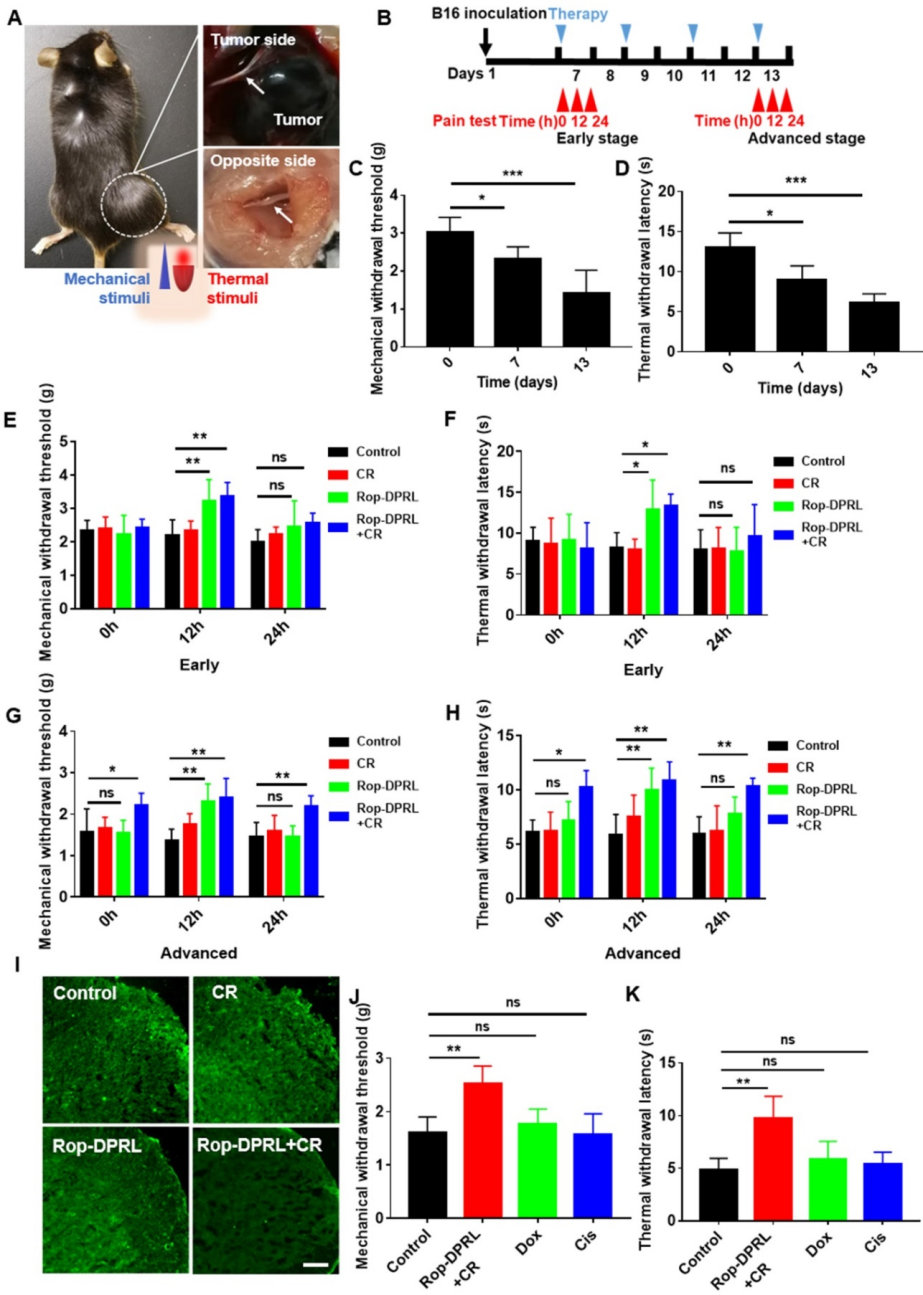
** Rop-DPRL combined with CR continuously and specifically relieves cancer pain. (A)** Bilateral dissected sciatic nerves (arrows) in a tumor-bearing mouse on day 13 post-inoculation. **(B)** Schematic diagram showing the experimental operation procedure. **(C, D)** Mechanical withdrawal threshold and thermal withdrawal latency were tested at day 0, day 7 and day 13. **(E-H)** Mechanical withdrawal threshold and thermal withdrawal latency were measured at early stage and advanced stage. Cancer pain was tested at 0 h, 12 h, and 24 h after Rop-DPRL administration. Early stage, day 7 after B16 inoculation; Advanced stage, day 13 after B16 inoculation. n= 5-7. **(I)** Immunofluorescent staining of Iba1 (a marker protein for microglia) in the spinal dorsal horn. n = 3. **(J, K)** Mechanical withdrawal threshold and thermal withdrawal latency in tumor-bearing mice. n = 5. Mechanical or Thermal pain was tested on day 13, at 24 h after Rop-DPRL+CR administration. CR: calorie restriction, Cis: cisplatin. Data are presented as the mean ± SEM, *p < 0.05, ** p < 0.01, *** p < 0.001, ns = no significant difference. Scale bar = 20 µm.

According to the volume of tumor, day 7 and day13 post-inoculation of B16 cell were defined as early and advanced stages of tumor growth, respectively. In advanced stage, the diameter of the tumor-sacral sciatic nerve was smaller than that on the contralateral side (Figure 6A), indicating that the cancer pain model was successfully established 38. Further, we continuously monitor the mechanical and thermal hyperalgesia of tumor-bearing mice (Figure 6B). The hind paw is one of the most commonly tested areas when dealing with rodent models of pain 57. Nociception on the hind paws of the model mouse in response to mechanical and thermal stimuli was tested using the electronic von Frey and Hargreaves thermal stimulator 24, which measured the ability of the mouse to withstand mechanical or thermal stimulation, respectively. The results showed that from early stage, mice began to show significant cancer pain (Figure 6C-D). Next, we detected the analgesic effect of Rop-DPRL at different stages. In the early stage, mechanical withdrawal threshold (MWT) and thermal withdrawal latency (TWL) of mice were significantly increased compared with control mice at 12 h after a single administration of Rop-DPRL, however, the changes were disappeared at 24 h (Figure 6E-F). These results indicated that a single injection of Rop-DPRL could relief cancer pain with its own analgestic ability in a short duration. In the advanced stage, separate administration of Rop-DPRL still significantly elevated the MWT and TWL with short duration (Figure 6G-H). Notably, mice under replicated combination therapy of Rop-DPRL and CR showed higher basal levels of MWT and TWL.24h after Rop-DPRL injection, significant increases in MWT and TWL were still detected in mice treated with combination therpy. (Figure 6G-H). Cancer pain is often accompanied by activation of microglia in the spinal dorsal horn 9, and the combination of Rop-DPRL with CR also inhibited the activation of microglia in the advanced stage (Figure 6I). These results indicated that the combination of Rop-DPRL and CR could continuously relieve cancer pain.

To investigate whether relief of cancer pain was due to tumor suppression, we evaluated the cancer pain in dox- and cisplatin-treated mice. Dox induced antitumor effect was similar to that observed in combination therapy with Rop-DPRL and CR, while cisplatin induced a milder antitumor effect (Figure S6). However, cisplatin and dox did not elevate MWT and TWL (Figure 6J-K). These results revealed that the combination therapy with Rop-DPRL and CR was specific in relieving advanced cancer pain. Moreover, there was no obvious difference between systemic and local treatment of Rop-DPRL based combination therapy on tumor suppression and pain relief (Figure S7).

### Reduction of VEGF-A expression contributes to relief of cancer pain by combination therapy

**Figure 7:**
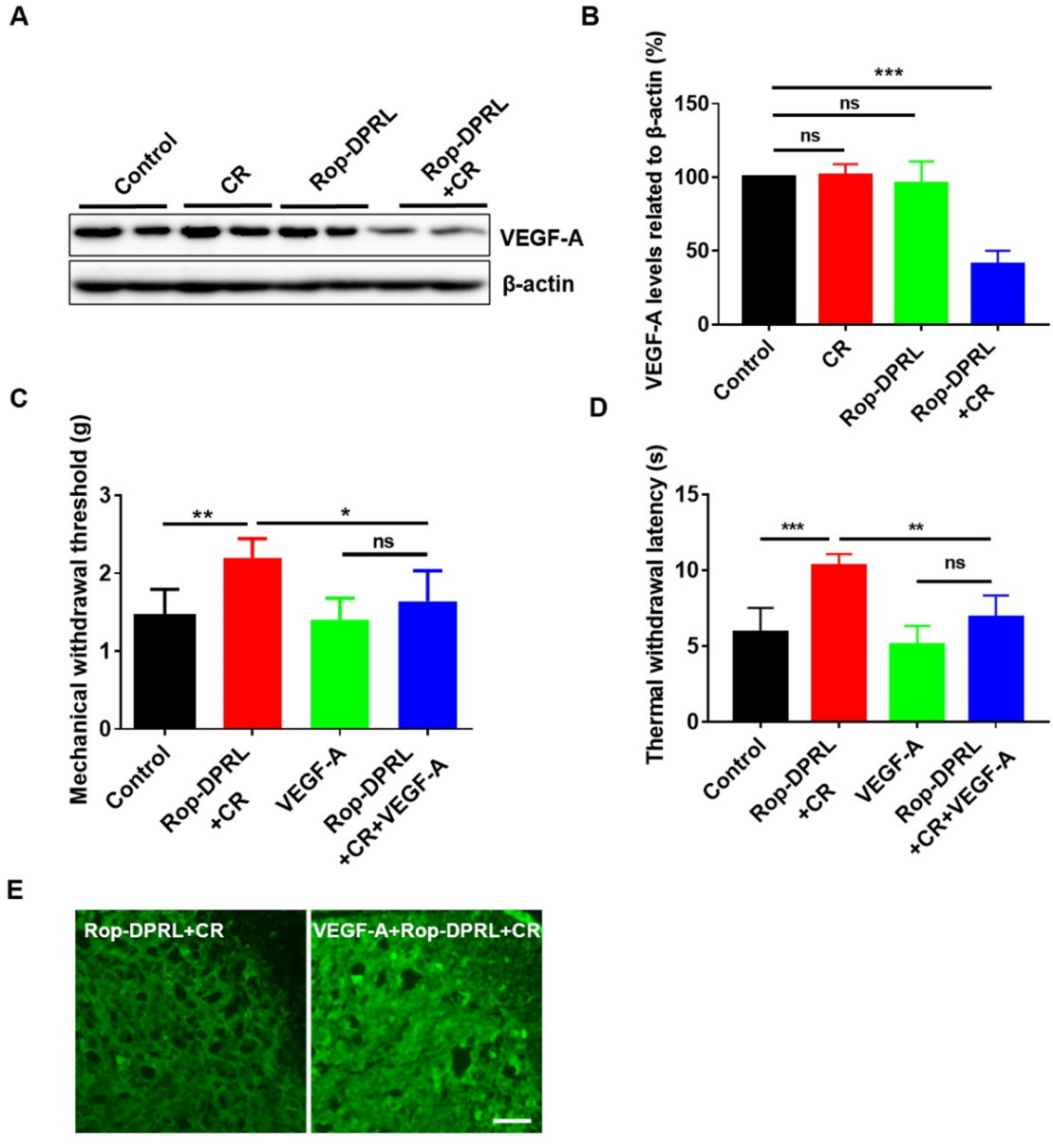
** Reduction of VEGF-A expression contributes to relief of cancer pain. (A)** Western blot and **(B)** statistical results of VEGF-A in tumor. **(C)** Mechanical paw withdrawal threshold of tumor-bearing mice. **(D)** Thermal paw withdrawal latency of tumor-bearing mice. **(E)** Immunofluorescent staining of Iba1 in the spinal dorsal horn. Tumor-bearing mice were treated with empty liposome, Rop-DPRL, CR, Rop-DPRL + CR, VEGF-A, or VEGF-A + Rop-DPRL + CR. VEGF-A was injected 3 h before pain test. Cancer pain was tested on day 13, at 24 h after Rop-DPRL administration. Data are presented as the mean ± SEM, n = 5, *p < 0.05, ** p < 0.01, *** p < 0.001, ns = no significant difference. Scale bar = 20 µm.

VEGFR1, which is expressed in peripheral nociceptors, mediates nociceptor excitability and pain hypersensitivity in human cancer and in mouse cancer models. VEGF-A is a specific ligand for VEGFR1, and injection of VEGF-A has been shown to induce nociceptive hypersensitivity in a mouse model of cancer pain 11. As such, we assessed the level of VEGF-A in our model. Interestingly, combination therapy with Rop-DPRL and CR significantly reduced VEGF-A level in mouse tumor tissue, whereas treatment with CR or Rop-DPRL alone did not alter VEGF-A level (Figure 7A-B). Furthermore, administration of VEGF-A abrogated the combination therapy-induced increases in MWT and TWL (Figure 7C-D) and blocked combination therapy-induced inhibition of microglial activation (Figure 7E). These results demonstrated that reduction of VEGF-A expression contributed to relief of cancer pain by combination therapy.

### Dual inhibition of the mTORC1/STAT3 and JAK2/STAT3 pathways mediate reduced expression of VEGF-A

**Figure 8:**
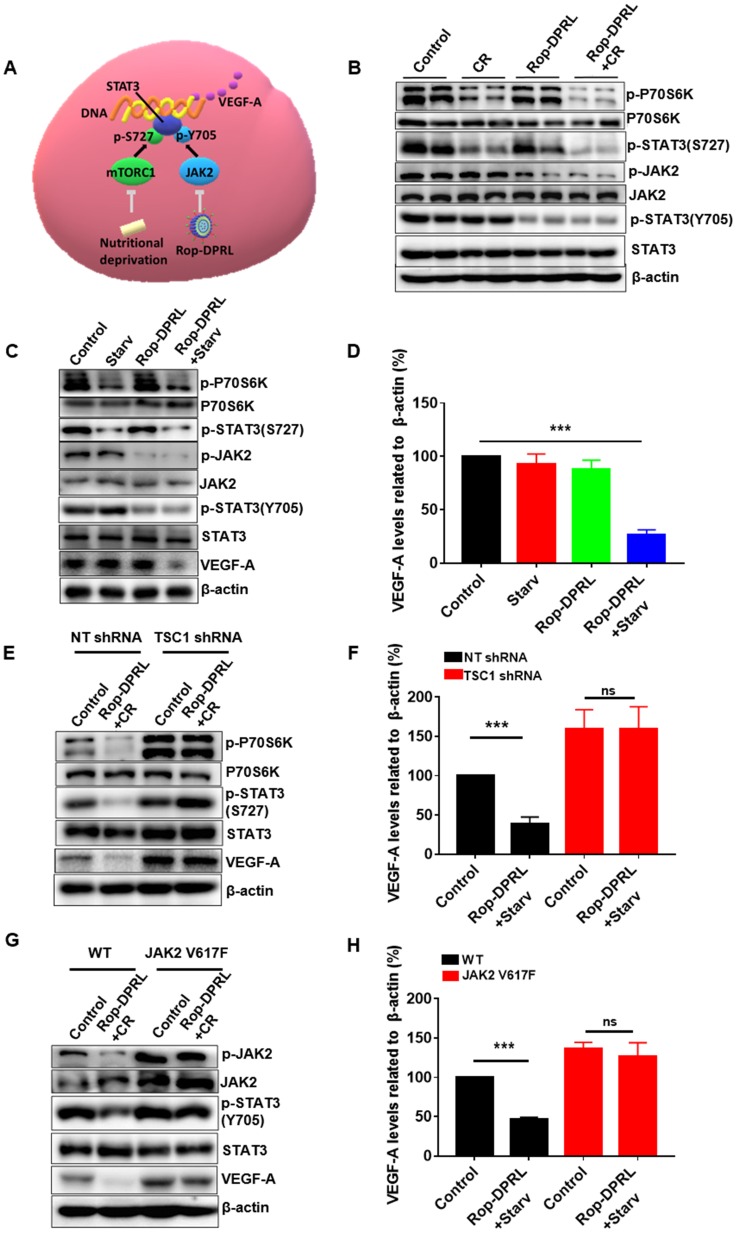
** Dual inhibition of the mTORC1/STAT3 and JAK2/STAT3 pathways results in reduced VEGF-A expression. (A)** An overview of mTORC1/STAT3 and JAK2/STAT3 pathways. **(B)** Western blot results of indicated protein in tumor. **(C, E, G)** Western blot results and **(D, F, H)** relative expression of VEGF-A in B16 cells. B16 cells were transduced with lentivirus containing empty vector, JAK2 V617F, shRNA targeting TSC1, or a non-target (NT) sequence. Cells were treated with Rop-DPRL, starvation or Rop-DPRL + starvation for 24 h. Data are presented as the mean ± SEM, *** p < 0.001, ns = no significant difference., starv: starvation.

Next, we tried to reveal the mechanism by which VEGF-A was reduced by combination therapy. VEGF-A is a target of STAT3, and mTORC1 and JAK2 promote activation of STAT3 through direct phosphorylation of Ser727 and Tyr705, respectively (Figure 8A). P70s6k is a major substrate of mTORC1, and its phosphorylation level reflects the activity of mTORC1. Our result showed that CR reduced p-P70s6k and p-STAT3 (Ser727) in tumor tissue (Figure 8B), which was consistent with previous studies 15. In addition, we found that separate Rop-DPRL treatment decreased phosphorylation of JAK2 and STAT3 (Tyr705) (Figure 8B). As such, the combination of CR and Rop-DPRL simultaneously inhibited the mTORC1/STAT3 and JAK2/STAT3 pathways (Figure 8B). Furthermore, we also confirmed dual inhibition of the mTORC1/STAT3 and JAK2/STAT3 pathways and reduction of VEGF-A in B16 cells following Rop-DPRL and CR treatment (Figure 8C-D). Then we try to verify the roles of the mTORC1 and JAK2 pathways in regulating VEGF-A level. Loss of TSC1 has been shown to increase the activity of mTORC1 58, 59. A valine-to-phenylalanine substitution at amino acid 617 results in constitutive tyrosine phosphorylation activity of JAK2 60, 61. In TSC1-knockdown and JAK2 V617F-overexpressing cells (Figure S8), combination treatment with starvation and Rop-DPRL did not reduce VEGF-A expression (Figure 8E-H). These results indicated that simultaneous inhibition of STAT3 at multiple sites may contribute to reduced VEGF-A expression.

## Conclusion

In summary, ropivacaine and ropivacaine- loaded liposomes (Rop-DPRL) were novelly found to damage autophagic degradation like autophagy inhibitors. Replicated administration of Rop-DPRL and CR could efficiently repress the development of tumor in a mice model of cancer pain which was induced by the tumor inoculated near the sciatic nerve. In addition, administration of Rop-DPRL could relieve cancer pain with its own analgestic ability in a short duration, while repeated administration of Rop-DPRL and CR resulted in continuous alleviation of cancer pain in advanced cancer by reducing VEGF-A. Further, dual inhibition of phosphorylation of STAT3 at Tyr705 and Ser727 by combination therapy contribute to the reduction of VEGF-A. This study suggests a new approach for simultaneously treating cancer and relieving cancer pain.

## Supplementary Material

Supplementary figures.

## Abbreviations

Rop-DPRL: ropivacaine-loaded liposomes
CR: calorie restriction
VEGF: vascular endothelial growth factor
STAT3: Signal transducer and activator of transcription 3
mTORC1: mammalian target of rapamycin complex 1
DLS: dynamic light scattering
Cath D: cathepsin D
CQ: chloroquine
MTT: 3-(4,5-dimethylthiazol-2-yl)-2,5-diphenyl tetrazolium bromide
TUNEL: terminal-deoxynucleoitidyl transferase mediated nick end labeling
MWT: mechanical withdrawal threshold
TWL: thermal withdrawal latency
