# Supplementary material for: Combination therapy with ropivacaine-loaded liposomes and nutrient deprivation for simultaneous cancer therapy and cancer pain relief
Source: Theranostics. 2020 Mar 26;10(11):4885–99. doi: 10.7150/thno.43932 (PMC7163441; doi:10.7150/thno.43932)
Supplement: Supplementary file 1 — Supplementary figures. [file thnov10p4885s1.pdf]

Figure S1

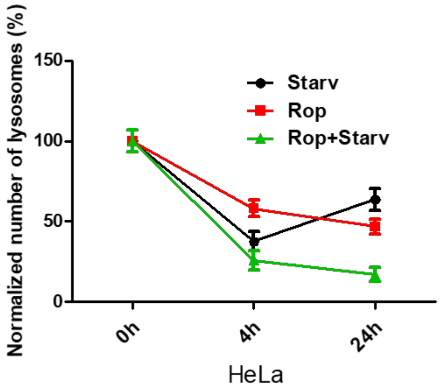

Figure S1. Statistical results of normalized number of lysosomes. HeLa cell were treated with ropivacaine for 0-24 h or starved for 0-24 h, then stained with 75 nM LysoTracker Red.

Figure S2

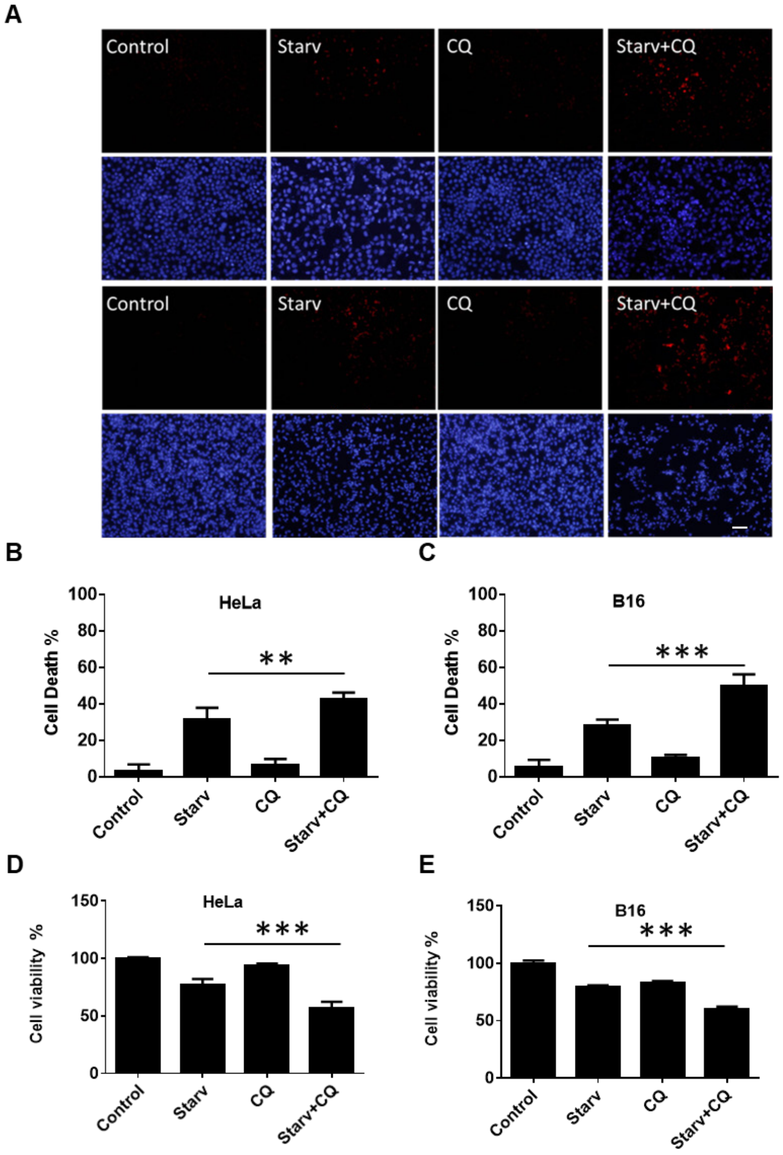

Figure S2. Autophagy promotes cancer cells survival in starvation environment. HeLa or B16 cells were treated with starvation or CQ (50 μM) for 24 h. (A) PI staining of HeLa or B16 cells. Nuclei were stained with Hoechst. (B, C) Cells from (A) were analyzed for percentage of dead cells. (D, E) Cell viability was determined using the MTT assay. Starv: starvation. Data are presented as the mean ± SEM, *$p < 0.05$, ** $p < 0.01$, ***$p < 0.001$, ns = no significant difference. Scale bar = 20 μm.

Figure S3

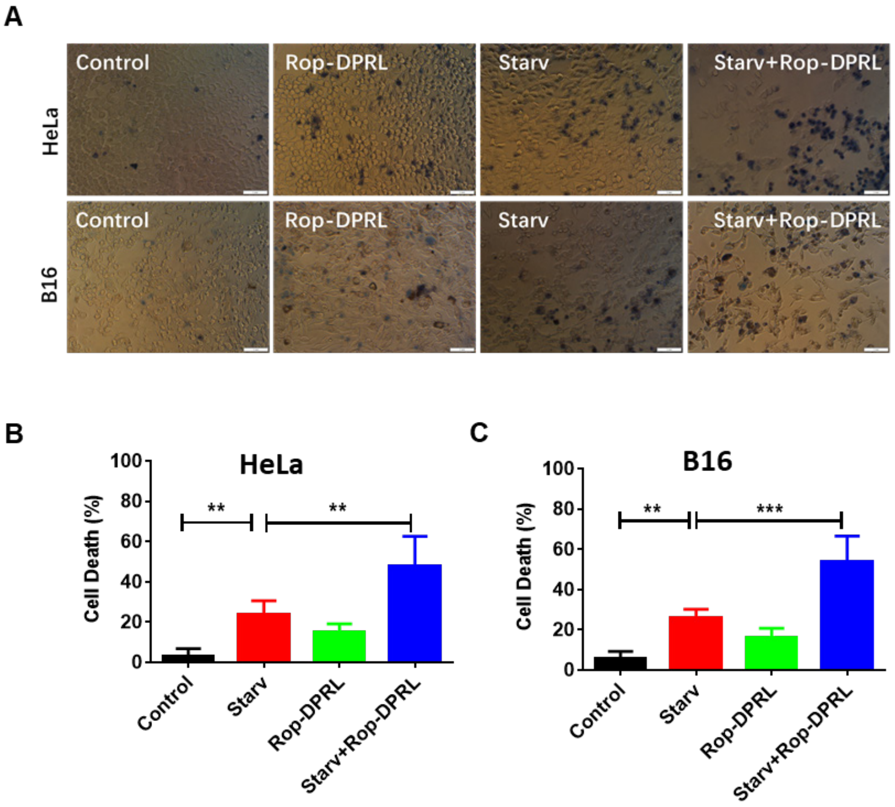

Figure S3. Rop-DPRL promote death of starved cancer cells. HeLa or B16 cells were treated with empty liposome, starvation or Rop-DPRL for 24 h. (A) Trypan blue staining and bright field image of HeLa and B16 cells. (B, C) Cells from (A) were analyzed for percentage of dead cells. Starv: starvation. Data are presented as the mean ± SEM, ** $p < 0.01$, ***$p < 0.001$, ns = no significant difference.

Figure S4

A

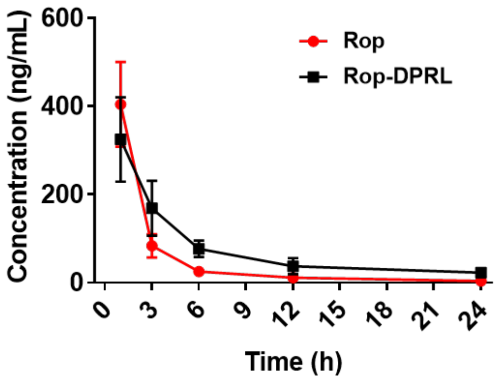

B

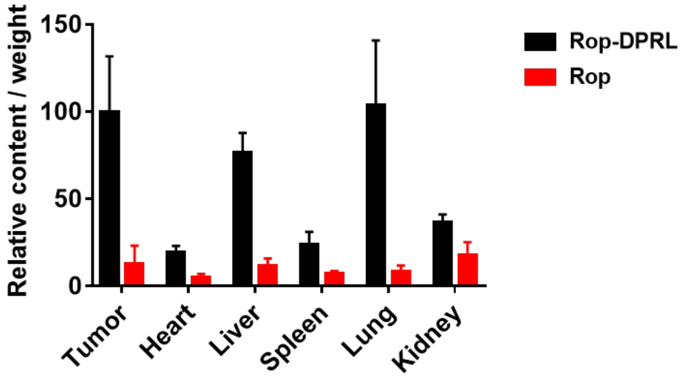

Figure S4. Pharmacokinetics and biodistribution of Rop and Rop-DPRL. (A) Plasma pharmacokinetic curves of Rop and Rop-DPRL. (B)Biodistribution of Rop and Rop-DPRL. Free ropivacaine (Rop) and Rop-DPRL with same ropivacaine concentration were injected into tumor-bearing mice. Mice blood were collected from orbital choroid plexus at 1 h, 3 h, 6 h, 12 h and 24 h. Another batch of mice were killed at 12 h, and tumor, heart, liver, spleen, lung and kidney were obtained. The content of ropivacaine in plasma and tissues were measured by HPLC according to Anesthesia & Analgesia,2014 ,118 (6): 1355-1362.

Figure S5

a

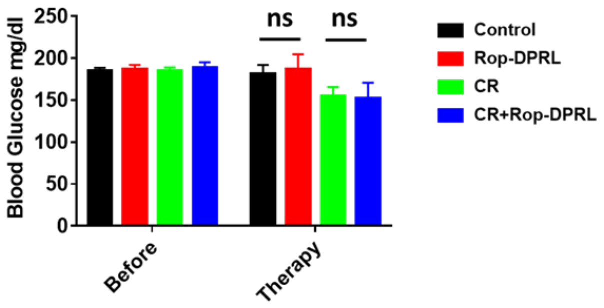

b

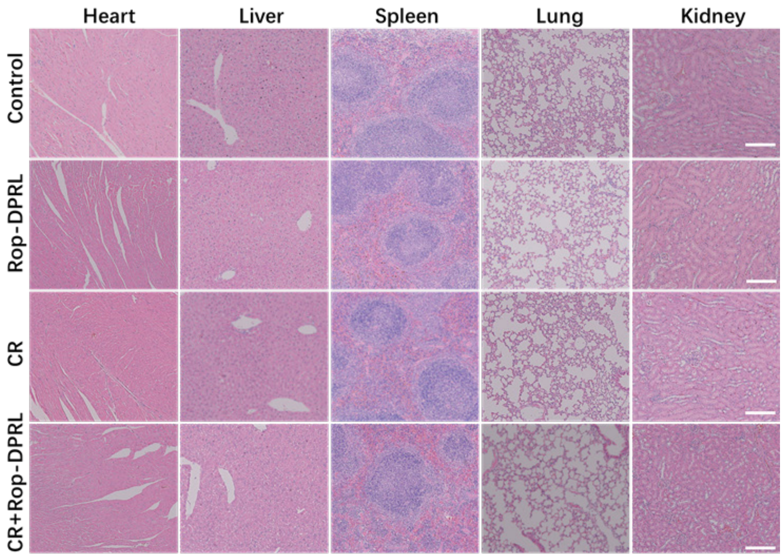

Supplementary Figure 5. Biosafety assessment of Rop-DPRL based therapy. (A) Blood glucose of mice. (B) HE staining images of major organs. Tumor-bearing mice were randomly grouped and received 4 times treatments with empty liposome, CR alone, Rop-DPRL alone, or Rop-DPRL combined with CR every other day. Food were given at 18:00. The blood glucose was measured before first therapy or at 24 h after the last therapy. All measurement and administration were carried out at the same time of day. Blood were collected by tail-cutting, and blood glucose were measured by glucometer (GA-2, SINOCARE). ns = no significant difference.

Figure S6

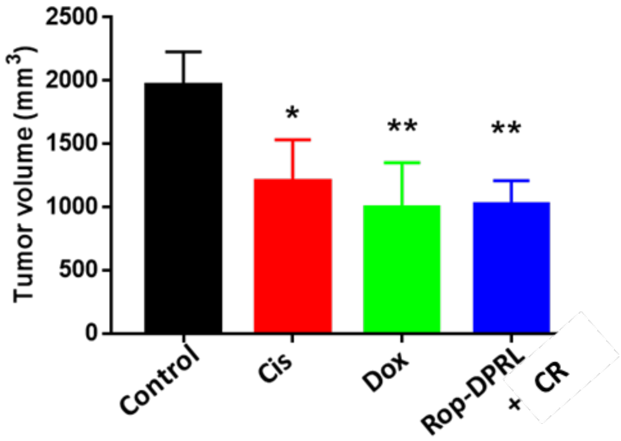

Figure S6. The volume of tumor. Mice were randomly divided into 4 groups. Mice were treated with empty liposome, Rop-DPRL+CR, cisplatin (4 mg/kg), or Dox (1.5 mg/kg). The volume of tumor was measured before killing mice.

Figure S7

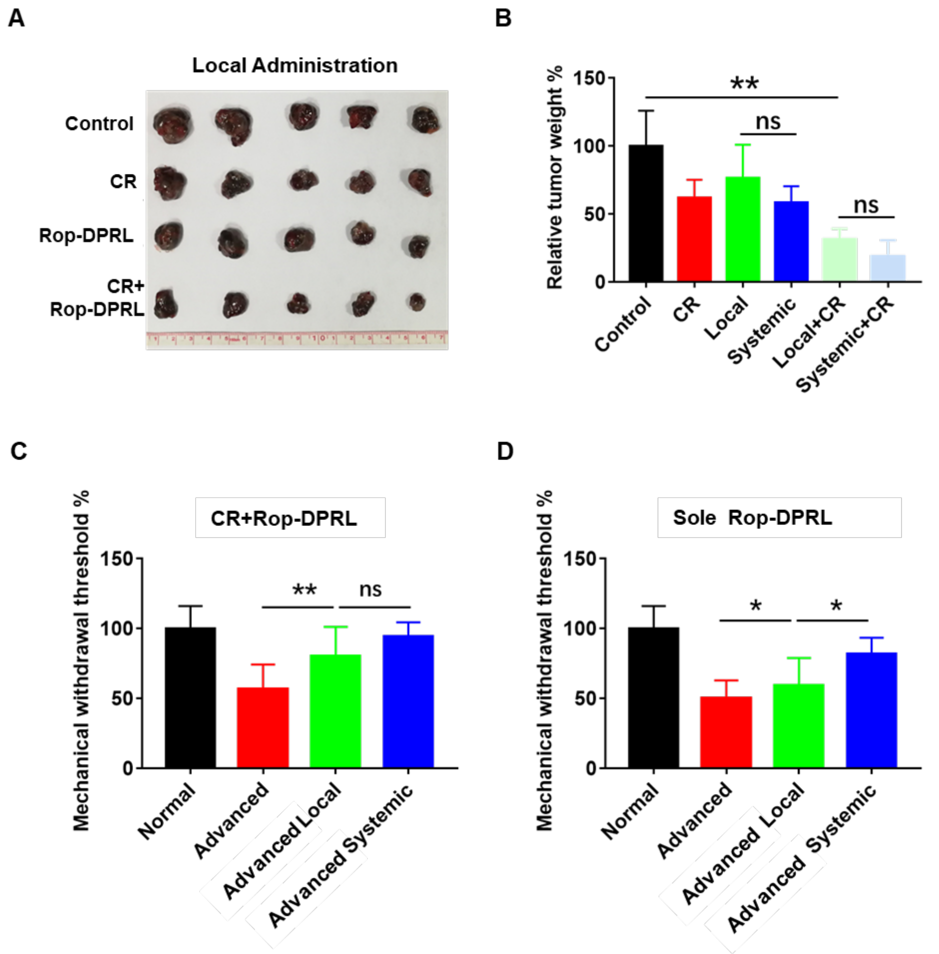

Supplementary Figure 7. Comparison between local and systemic administration. (A) Photo of tumors from mice under local administration. (B) Relative tumor weight. (C) Mechanical withdrawal threshold of mice under the treatment of CR combined with Rop-DPRL. Mechanical withdrawal threshold was measured at advanced stage. (D) Mechanical withdrawal threshold of mice under the sole treatment of Rop-DPRL. Tumor-bearing mice were randomly grouped and received treatments with empty liposome, CR alone, Rop-DPRL alone, or Rop-DPRL combined with CR. For local administration, empty liposomes and Rop-DPRL were injected to the tumor tissue every other day. For systemic administration, liposomes and Rop-DPRL were intraperitoneally injected every other day. Mice were treated 4 times before being killed. 24 h after the last treatment, the mechanical pain of mice was tested. Tumor was taken out and weighted. *$p < 0.05$, ** $p < 0.01$, ns = no significant difference.

Figure S8

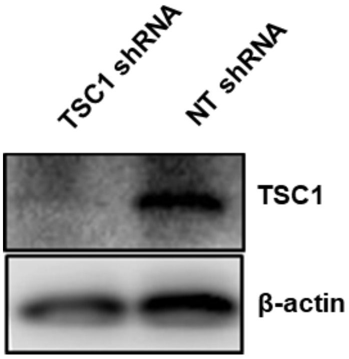

Figure S8. Western blot results of TSC1 and β-actin. B16 cells were transduced with lentivirus containing empty vector or shRNA targeting TSC1.
